# Kidney Transplantation in Children and Adolescents With C3 Glomerulopathy or Immune Complex Membranoproliferative Glomerulonephritis: An International Survey of Current Practice

**DOI:** 10.1111/petr.70048

**Published:** 2025-02-24

**Authors:** Christian Patry, Nicholas J. A. Webb, Matthias Meier, Lars Pape, Alexander Fichtner, Britta Höcker, Burkhard Tönshoff

**Affiliations:** ^1^ Department of Pediatrics I, University Children's Hospital Heidelberg, Medical Faculty Heidelberg University Heidelberg Germany; ^2^ Novartis Pharma AG Basel Switzerland; ^3^ Department of Paediatrics and Adolescent Medicine II Essen University Hospital Essen Germany

**Keywords:** complement 3 glomerulopathy, pediatric kidney transplantation, primary immune‐complex membranoproliferative glomerulonephritis, recurrence post‐transplant, survey

## Abstract

**Background:**

Approximately 50% of patients with chronic kidney disease due to C3 glomerulopathy (C3G) or primary immune‐complex membranoproliferative glomerulonephritis (IC‐MPGN) will require dialysis and/or kidney transplantation (KTx) within the first 10 years of disease onset. Currently, there are no guidelines regarding the indications for KTx or post‐transplant management.

**Methods:**

We therefore initiated an international online survey via Survey Monkey on C3G and IC‐MPGN in children with CKD stage 5. All KTx centers of the European Society for Paediatric Nephrology (ESPN) were invited to participate in the survey, which was conducted from August 23 to November 25, 2023.

**Results:**

Sixty‐five (63%) of the centers (*n* = 103) participated. Twenty‐six percent had made at least one decision against living donation for a child with C3G or IC‐MPGN. The main reason for 88.2% of these decisions was concern about the recurrence of the underlying disease in any potential transplant. Eighty‐eight percent indicated deceased donation as an option; 12% decided not to proceed with transplantation at all. Regarding KTx decision‐making or management, none of them referred to an existing recommendation by any national or regional guideline. For the recurrence of C3G or IC‐MPGN post‐transplant, eculizumab treatment was suggested by 60% of respondents.

**Conclusion:**

This survey shows a considerable reluctance of pediatric nephrologists to list patients with CKD stage 5 due to C3G or IC‐MPGN for living donor kidney transplantation. This decision is mainly based on the fear of recurrence of the underlying disease combined with the lack of reliable treatment options. This limited access of affected patients to the best treatment option for kidney failure requires further action.

## Graphical abstract

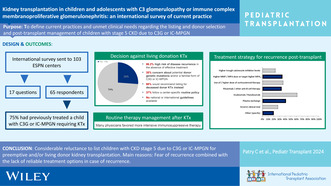

## 1 Introduction

Complement 3 glomerulopathy (C3G), an ultra‐rare chronic kidney disease, is associated with dysregulation of the alternative complement pathway (AP) in plasma and the glomerular microenvironment, thereby resulting in the accumulation of C3 and its cleavage products in the glomerulus [1, 2]. C3G is caused by acquired and/or genetic abnormalities affecting the complement pathway, such as nephritic factors, or genetic variants in key AP complement genes. The overall prognosis is poor, with approximately 40%–50% of patients progressing to kidney failure within 10 years of diagnosis. There are currently no approved treatments for C3G, and in particular none that specifically target the underlying complement‐mediated pathophysiology. One of the major challenges in the treatment of patients with C3G and IC‐MGPN is the high rate of disease recurrence in the transplanted kidney (40%–60%), often leading to graft loss and referral to maintenance dialysis [3, 4].

Immune complex membranoproliferative glomerulonephritis (IC‐MPGN) is an ultra‐rare, rapidly progressive kidney disease that may be idiopathic (primary IC‐MPGN) or secondary to chronic infections and autoimmune diseases [5]. Although IC‐MPGN is a distinct disease according to the current classification, it shares important similarities with C3G, which is also typically characterized by membranoproliferative or mesangioproliferative histopathologic patterns. Similar to C3G, there is clear evidence of dysregulation of the complement alternative pathway playing a central role in the disease pathogenesis [6]. Like C3G, IC‐MPGN is commonly diagnosed in childhood and adolescence, with a median age at diagnosis of approximately 21 years [7, 8] and is also characterized by a high risk of progression to kidney failure [6, 7]. Patients with IC‐MPGN who undergo KTx may also experience disease recurrence. Although there is some evidence that post‐transplant recurrence may be less common in IC‐MPGN than in C3G, outcomes are nevertheless poor in both [9]. Patients who experience disease recurrence are at high risk for subsequent kidney graft failure.

There is considerable uncertainty and variability among pediatric nephrologists regarding listing for transplantation and optimal post‐transplant management in the absence of international consensus guidelines. Therefore, the Transplantation Working Group of the European Society for Paediatric Nephrology (ESPN) and the Cooperative European Paediatric Renal Transplant Initiative (CERTAIN) research network developed a questionnaire on center practice in the management of patients with C3G or IC‐MPGN with stage 5 chronic kidney disease (CKD) or kidney transplant and recurrent disease. The purpose of this survey was to define current practices and unmet clinical needs regarding the listing and donor selection of pediatric patients with C3G or IC‐MPGN and stage 5 CKD, as well as post‐transplant management.

## 2 Methods

We conducted a questionnaire‐based cross‐sectional survey performed on behalf of the ESPN working group “Transplantation” and the CERTAIN research network. An invitation to participate in this survey was sent via email to all active ESPN members (*n* = 391). The invitation included a brief overview of the study, the investigators involved, and a link to the questionnaire. Currently, approximately 103 centers in the ESPN perform kidney transplantation in children and adolescents. The survey was carried out between August 23, 2023, and November 25, 2023. An email invitation and one reminder with a summary of the project and a personal link to an Internet questionnaire service provider (https://es.surveymonkey.com/) were sent to the ESPN members. The survey consisted of 17 questions addressing listing for deceased donation, living donation, preemptive KTx, and choice of immunosuppressive therapy post‐transplant and in case of recurrence. The study was approved by the council of the ESPN. Requests for authorization by the ethics committees of each center were not considered necessary because this was a survey that simply collected the experience and practices of the physicians, and it did not involve approaching patients directly or seeking any patient‐specific data. A database including all the responses of the participants was provided by the Internet questionnaire service for further analysis.

## 3 Results

A total of 65 ESPN physicians participated in the survey. Members were asked to provide only one representative response for each pediatric kidney transplant center. Therefore, it can be assumed that 65 of 103 (63%) pediatric kidney transplant centers within the ESPN participated in this survey.

Of the 65 respondents, 49 (75%) indicated that they had previously been responsible for the management of a child or adolescent with C3G or IC‐MPGN who required kidney transplantation. Of the 65 respondents, 8 (12%) reported that they had on at least one occasion decided not to perform a kidney transplant in a child or adolescent with renal failure secondary to C3G or IC‐MPGN; they justified this decision on the basis of the high risk of recurrent disease in the transplant for which no effective treatment was currently available. They also indicated that this was routine policy for all children and adolescents with C3G or IC‐MPGN at their respective transplant centers, but that there was no specific written local or national guideline regarding listing for kidney transplantation in this population.

Regarding the selection of a potential kidney donor, 17 of the 65 (26%) responded that they had on at least one occasion decided not to use a potentially suitable living related or unrelated donor because of a diagnosis of C3G or IC‐MPGN. The specific reason for not performing a living donor kidney transplant in these patients was the high risk of disease recurrence in the absence of effective treatment for the majority (88.2%) of respondents, and concern about potential donor genetic mutations and/or a familial form of C3G or IC‐MPGN for 35.3%. In such a situation, the majority (88%) would recommend listing for deceased donor transplantation only; 12% decided not to proceed with transplantation at all. However, there was some flexibility in decision‐making, as 62.5% indicated that this was not a routine policy for all children and adolescents with C3G or IC‐MPGN at their transplant center. All respondents confirmed that there was no specific written local or national guideline regarding the use of living donors for kidney transplantation in children and adolescents with C3G or IC‐MPGN.

Regarding preemptive (pre‐dialysis) kidney transplantation in these patients, 9% stated that they have on at least one occasion decided not to perform a preemptive (pre‐dialysis) transplantation because of a high risk of recurrent disease with no effective treatment available; the majority (67%) would recommend listing for deceased donor transplantation only; 33% responded that they would not perform transplantation in such a situation.

**FIGURE 1:**
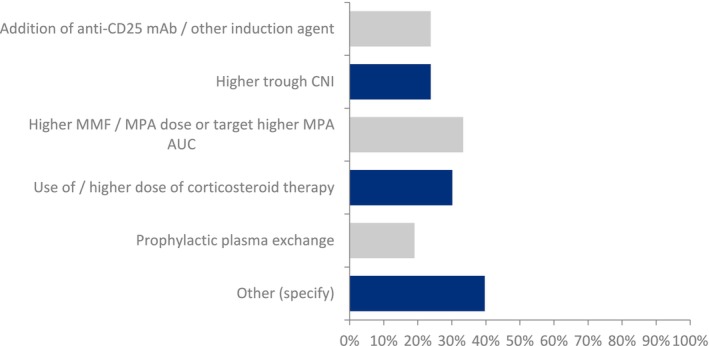
Responses to the survey question: Do you alter your routine post‐transplant immunosuppressive protocol for children/adolescents with C3G/IC‐MPGN undergoing transplantation in your transplantation center? AUC, area under the curve; CNI, calcineurin inhibitor; mAb, monoclonal antibody; MMF, mycophenolate mofetil; MPA, mycophenolic acid.

**FIGURE 2:**
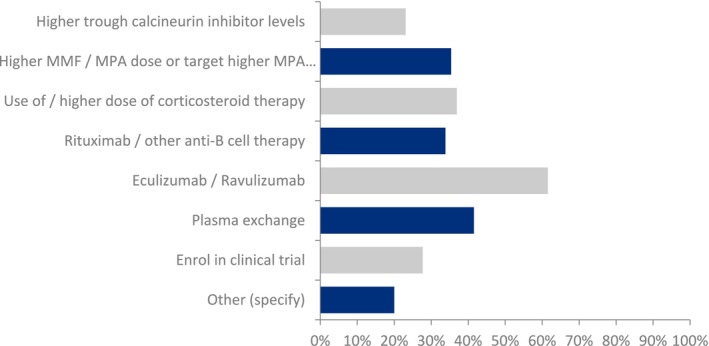
Responses to the survey question: What is your treatment strategy in patients who develop recurrent C3G/IC‐MPGN following kidney transplantation? Abbreviations: MMF, mycophenolate mofetil; MPA, mycophenolic acid.

The next question related to the routine post‐transplant immunosuppressive protocol for children and adolescents with C3G or IC‐MPGN undergoing kidney transplantation. Many physicians favored more intensive immunosuppressive therapy, but the choice of immunosuppressant varied (Figure 1). The most popular approach was the intensification of MMF therapy or MPA exposure. Regarding the treatment strategy for patients who develop recurrent C3G or IC‐MPGN, complement factor 5 inhibition with either eculizumab or ravulizumab was the preferred approach, followed by plasma exchange and the intensification of steroid or MMF/MPA‐based maintenance immunosuppressive therapy (Figure 2). Interestingly, a significant number of physicians (28%) would wish to enroll these patients in a clinical trial, reflecting the growing interest and awareness of new medications in this disease area.

## 4 Discussion

The results of this survey reflect the medical dilemma faced by physicians treating patients with kidney failure due to C3G or IC‐MPGN. Disease recurrence occurs in 40%–50% of patients at 14–28 months post‐transplant, and kidney failure has been reported in 15%–47% of such patients, with a median time to graft failure of approximately 4–6 years [3, 4]. There is currently no FDA‐ or EMA‐approved therapy for these conditions either pre‐ or post‐transplant [10, 11]. As a result, 12% of respondents to this survey would not recommend kidney transplantation for patients with C3G or IC‐MPGN, despite the fact that kidney transplantation is the treatment of choice for kidney failure in children, as it is associated with significantly lower patient morbidity and mortality and significantly improved quality of life compared to chronic dialysis therapy. The lack of a written local or national guidelines for listing for kidney transplantation was cited by all survey respondents.

Regarding the selection of a potential kidney donor, 26% were opposed to living donor kidney transplantation due to the risk of recurrence of the primary kidney disease or potential donor genetic mutations and/or a familial form of C3G or IC‐MPGN. For other underlying kidney diseases with a high risk of recurrence post‐transplant, such as primary immune‐mediated focal segmental glomerulosclerosis, physicians also have some reluctance to consider living kidney donation [12]. On the other hand, the risk of recurrence of C3G or IC‐MGPN in kidney transplantation (40%–60%) is not 100%, so the individual decision to accept a living donor for transplantation could be considered reasonable.

Preemptive kidney transplantation is generally considered to be the treatment of choice for pediatric patients with kidney failure because it is associated with both better graft and patient survival than transplantation after maintenance dialysis therapy [13]. The reluctance to offer preemptive kidney transplantation to a patient with a high risk of recurrence of the underlying disease is widespread. The underlying consideration is that a short period of time between an active autoimmune glomerulonephritis in the patient's own kidneys and a kidney transplantation may be associated with a higher risk of recurrence. For autoimmune glomerulonephritides with a high risk of recurrence after transplantation, such as C3G or lupus nephritis, many clinicians therefore wait for approximately 2 years after the patient has reached CKD stage 5 in the hope that the activity of the autoimmunity and thus the risk of recurrence will decrease over time.

Regarding the routine post‐transplant immunosuppressive protocol, the majority of respondents favored more intense immunosuppressive therapy, specifically more intense MMF‐based therapy, given the approximately 50% risk of relapse. This is not entirely surprising since MMF, in combination with glucocorticoids, is the currently recommended therapy for C3G in the native kidneys according to the recent KDIGO guidelines [14]. The wide variability in the responses (Figure 1) reflects the fact that there is no established immunosuppressive regimen recommended by guidelines to prevent the recurrence of C3G in kidney transplants.

For patients who have developed recurrent C3G or IC‐MPGN post‐transplant, the preferred treatment strategy was complement factor 5 inhibition with either eculizumab or ravulizumab (Figure 2); but again, various other treatment strategies were also considered. In a systematic review of the treatment of C3G in adult kidney transplant recipients, Suarez et al. reported 12 studies, including seven cohort studies and five case series, comparing the treatment of patients with eculizumab, rituximab, plasmapheresis, and no treatment [15]. The total number of patients diagnosed with post‐transplant C3G was *n* = 122. They observed that eculizumab reduced graft loss compared to rituximab and plasmapheresis (33% eculizumab, 42% plasmapheresis, 81% rituximab). The response to eculizumab was related to initial soluble C5b‐9 levels: patients with higher levels of C5b‐9 showed a better response to eculizumab. A recent retrospective case series reported on 8 patients with post‐transplant recurrence of C3G; four of eight patients (50%) treated with eculizumab showed complete response [16]. However, after a median of 27 months, five kidney transplant recipients experienced graft loss despite eculizumab use in three of them. The authors concluded that prognosis is still quite dismal in kidney transplant recipients with C3G recurrence despite eculizumab use, presumably because these patients have a quiescent progressive course instead of a crescentic rapidly progressive disease in native kidneys, which responds better to eculizumab [17]. Regarding pediatric kidney transplant recipients, we recently reported a multicenter longitudinal cohort study of the CERTAIN research network, in which we compared the post‐transplant outcomes of patients with C3G (*n* = 17) or IC‐MPGN (*n* = 3) with a matched case–control group [4]. Complement‐targeted therapy with eculizumab, either as prophylaxis or treatment, did not appear to be effective.

While the results of this survey reflect the current challenges in managing kidney transplantation in pediatric patients with C3G or IC‐MPGN, the field of complement‐targeted therapies for kidney diseases is rapidly evolving. Recent research in the treatment of C3G and IC‐MPGN, including phase 2 and phase 3 clinical trials, has focused on two promising agents: iptacopan, a factor B inhibitor, and pegcetacoplan, a targeted C3 and C3b inhibitor. Both agents have demonstrated efficacy and safety in addressing complement‐mediated pathophysiology in both native C3G and in patients with C3G recurrence post‐transplant. A phase 2 trial demonstrated that a 12‐week course of iptacopan reduced proteinuria by 45% in native C3G and reduced C3 staining in those with recurrent disease [18]. In the subsequent phase 3 double‐blind APPEAR trial, treatment with iptacopan reduced proteinuria by 35.1% compared with placebo and improved renal outcomes in adult patients with native C3G [19]. In addition, a case study of a transplant recipient with recurrent dense deposit disease reported successful treatment with iptacopan when conventional therapies had failed [20].

Direct C3 inhibition with pegcetacoplan offers another promising therapeutic option to control complement‐mediated renal pathology. The phase 2 NOBLE trial demonstrated a significant reduction in C3c staining accompanied by a 56.4% reduction in proteinuria following 52 weeks of pegcetacoplan therapy in patients with recurrent post‐transplant C3G or IC‐MPGN [21]. The phase 3 VALIANT study confirmed this efficacy in adults and adolescents with both native and recurrent C3G or primary IC‐MPGN, with a 68.3% relative reduction in proteinuria compared with placebo following 26 weeks of treatment, along with stabilization of eGFR and reduction in C3c staining [22]. Both the VALIANT and APPEAR studies have extension studies to evaluate long‐term efficacy and safety [23, 24]. Given the encouraging results demonstrated in the treatment of recurrent disease, there is also the potential to use these agents prophylactically to prevent recurrence from occurring. While regulatory approval for the use of these agents post‐transplant is pending, their availability to kidney transplant recipients may increase the willingness to accept living donor kidney transplantation for patients with C3G or IC‐MPGN in the future.

In conclusion, this survey shows a considerable reluctance of pediatric nephrologists to list patients with CKD stage 5 due to C3G or IC‐MPGN for preemptive and/or living donor kidney transplantation. This decision is based mainly on the fear of recurrence of the underlying disease combined with the lack of reliable treatment options. This limited access of affected patients to the best treatment option for kidney failure will hopefully change in the future with the approval of effective drugs for the prophylaxis and treatment of recurrent disease after transplantation [10, 25, 26].

## Conflicts of Interest

Nicholas J. A. Webb and Matthias Meier were employees of Novartis Pharma AG, Basel, Switzerland, at the time of this study; Nicholas J. A. Webb is currently an employee of Swedish Orphan Biovitrum (Sobi) AG, Basel, Switzerland. Burkhard Tönshoff has received research grants from Novartis, Astellas, Takeda, and Chiesi, and consulting fees from Bristol‐Myers Squibb, CSL Behring Biotherapies for Life, Vifor, and Chiesi.

## Data Availability

The data that support the findings of this study are available from the corresponding author upon reasonable request.

## References

[1] F. Caravaca‐Fontán , L. Lucientes , T. Cavero , and M. Praga , “Update on C3 Glomerulopathy: A Complement‐Mediated Disease,” Nephron 144 (2020): 272–280. 32369815 10.1159/000507254

[2] S. Sethi , M. Haas , G. S. Markowitz , et al., “Mayo Clinic/Renal Pathology Society Consensus Report on Pathologic Classification, Diagnosis, and Reporting of GN,” Journal of the American Society of Nephrology 27 (2016): 1278–1287. 26567243 10.1681/ASN.2015060612 PMC4849835

[3] S. Obata , P. A. S. Vaz de Castro , L. V. Riella , and P. Cravedi , “Recurrent C3 Glomerulopathy After Kidney Transplantation,” Transplantation Reviews 38 (2024): 100839. 38412598 10.1016/j.trre.2024.100839

[4] C. Patry , N. J. A. Webb , M. Feißt , et al., “Kidney Transplantation in Children and Adolescents With C3 Glomerulopathy or Immune Complex Membranoproliferative Glomerulonephritis: A Real‐World Study Within the CERTAIN Research Network,” Pediatric Nephrology 39, no. 12 (2024): 3569–3580, 10.1007/s00467-024-06476-5. 39110227 PMC11511764

[5] S. H. Nasr , A. M. Valeri , G. B. Appel , et al., “Dense Deposit Disease: Clinicopathologic Study of 32 Pediatric and Adult Patients,” Clinical Journal of the American Society of Nephrology 4 (2009): 22–32. 18971369 10.2215/CJN.03480708 PMC2615696

[6] E. K. S. Wong , K. J. Marchbank , H. Lomax‐Browne , et al., “C3 Glomerulopathy and Related Disorders in Children Etiology‐Phenotype Correlation and Outcomes,” Clinical Journal of the American Society of Nephrology 16 (2021): 1639–1651. 34551983 10.2215/CJN.00320121 PMC8729419

[7] P. Iatropoulos , E. Daina , M. Curreri , et al., “Cluster Analysis Identifies Distinct Pathogenetic Patterns in c3 Glomerulopathies/Immune Complex–Mediated Membranoproliferative GN,” Journal of the American Society of Nephrology 29 (2018): 283–294. 29030465 10.1681/ASN.2017030258 PMC5748907

[8] N. Garam , Z. Prohászka , Á. Szilágyi , et al., “Validation of Distinct Pathogenic Patterns in a Cohort of Membranoproliferative Glomerulonephritis Patients by Cluster Analysis,” Clinical Kidney Journal 13 (2019): 225–234. 32296528 10.1093/ckj/sfz073 PMC7147314

[9] A. Servais , L. H. Noël , L. T. Roumenina , et al., “Acquired and Genetic Complement Abnormalities Play a Critical Role in Dense Deposit Disease and Other C3 Glomerulopathies,” Kidney International 82 (2012): 454–464. 22456601 10.1038/ki.2012.63

[10] B. Tarragon Estebanez and A. S. Bomback , “C3 Glomerulopathy: Novel Treatment Paradigms,” Kidney International Reports 9, no. 3 (2023): 569–579. 38481517 10.1016/j.ekir.2023.12.007 PMC10927479

[11] G. Bartoli , A. Dello Strologo , G. Grandaliano , and F. Pesce , “Updates on C3 Glomerulopathy in Kidney Transplantation: Pathogenesis and Treatment Options,” International Journal of Molecular Sciences 25, no. 12 (2024): 6508. 38928213 10.3390/ijms25126508 PMC11204074

[12] L. T. Weber , B. Tönshoff , R. Grenda , et al., “Clinical Practice Recommendations for Recurrence of Focal and Segmental Glomerulosclerosis/Steroid‐Resistant Nephrotic Syndrome,” Pediatric Transplantation 25, no. 3 (2021): e13955. 33378587 10.1111/petr.13955

[13] R. Rana Magar , S. Knight , J. Stojanovic , et al., “Is Preemptive Kidney Transplantation Associated With Improved Outcomes When Compared to Non‐preemptive Kidney Transplantation in Children? A Systematic Review and Meta‐Analysis,” Transplant International 35 (2022): 10315. 35368639 10.3389/ti.2022.10315 PMC8967954

[14] B. H. Rovin , S. G. Adler , J. Barratt , et al., “KDIGO 2021 Clinical Practice Guideline for the Management of Glomerular Diseases,” Kidney International 100, no. 4S (2021): S1–S276. 34556256 10.1016/j.kint.2021.05.021

[15] M. L. Gonzalez Suarez , C. Thongprayoon , P. Hansrivijit , et al., “Treatment of C3 Glomerulopathy in Adult Kidney Transplant Recipients: A Systematic Review,” Medical Sciences 8, no. 4 (2020): 44. 33096866 10.3390/medsci8040044 PMC7712822

[16] S. Mirioglu , R. H. Hocaoglu , A. Velioglu , et al., “Prognosis is Still Poor in Patients With Posttransplant C3 Glomerulopathy Despite Eculizumab Use,” Clinical Kidney Journal 17, no. 7 (2024): sfae190. 39021814 10.1093/ckj/sfae190 PMC11252667

[17] M. Le Quintrec , A. L. Lapeyraque , A. Lionet , et al., “Patterns of Clinical Response to Eculizumab in Patients With C3 Glomerulopathy,” American Journal of Kidney Diseases 72, no. 1 (2018): 84–92. 29429752 10.1053/j.ajkd.2017.11.019

[18] E. Wong , C. Nester , T. Cavero , et al., “Efficacy and Safety of Iptacopan in Patients With C3 Glomerulopathy,” Kidney International Reports 8 (2023): 2754–2764. 38106570 10.1016/j.ekir.2023.09.017 PMC10719607

[19] C. M. Nester , R. J. Smith , D. Kavanagh , et al., “12‐Month Results From the Phase 3 APPEAR‐C3G Study,” Journal of the American Society of Nephrology, Kidney Week Edition, Abstract Supplement 35, no. 10S (2024), 10.1681/ASN.20241h4rz9f5.

[20] K. S. Wei , C. M. Nester , and N. Khan , “Novel Therapy for Recurrent Dense Deposit Disease in an Adolescent Kidney Transplant Recipient,” Journal of the American Society of Nephrology, Kidney Week Edition, Abstract Supplement 35, no. 10S (2024), 10.1681/ASN.20241h4rz9f5.

[21] A. Java , A. S. Bomback , D. Kavanagh , et al., “Pegcetacoplan for Post‐Transplant Recurrent C3 Glomerulopathy (C3G) or Immune Complex Membranoproliferative Glomerulonephritis (IC‐MPGN) in NOBLE: 52‐Week Patient Evolution,” Journal of the American Society of Nephrology, Kidney Week Edition, Abstract Supplement 35, no. 10S (2024), 10.1681/ASN.20241h4rz9f5.

[22] C. M. Nester , A. S. Bomback , M. G. Ariceta Iraola , et al., “VALIANT: A Randomized, Multicenter, Double‐Blind, Placebo (PBO)‐Controlled, Phase 3 Trial of Pegcetacoplan for Patients With Native or Post‐Transplant Recurrent Glomerulopathy (C3G) or Primary Immune Complex Membranoproliferative Glomerulonephritis (IC‐MPGN),” Journal of the American Society of Nephrology, Kidney Week Edition, Abstract Supplement 35, no. 10S (2024), 10.1681/ASN.20241h4rz9f5.

[23] C. M. Nester , M. G. Ariceta Iraola , A. Mukherjee , L. Li , J. Szamosi , and L. López Lázaro , “Long‐Term Safety and Efficacy of Pegcetacoplan in Patients With C3 Glomerulopathy or Primary Immune‐Complex Membranoproliferative Glomerulonephritis: The Long‐Term VALE Extension Study,” Journal of the American Society of Nephrology, Kidney Week Edition, Abstract Supplement 35, no. 10S (2024), 10.1681/ASN.20241h4rz9f5.

[24] A. S. Bomback , D. Kavanagh , M. Vivarelli , et al., “Alternative Complement Pathway Inhibition With Iptacopan for the Treatment of C3 Glomerulopathy – Study Design of the APPEAR C3G Trial,” Kidney International Reports 7 (2022): 2150–2159. 36217526 10.1016/j.ekir.2022.07.004 PMC9546729

[25] M. C. Mancuso , A. Mastrangelo , L. Dato , S. Verdesca , M. Cugno , and G. Ardissino , “Efficacy of Pegcetacoplan in Children With C3 Glomerulopathy,” Journal of the American Society of Nephrology, Kidney Week Edition, Abstract Supplement 35, no. 10S (2024), 10.1681/ASN.20241h4rz9f5.

[26] V. J. Escudero‐Saiz , Á. Gonzalez , A. García‐Herrera , et al., “Factor B Inhibition With Iptacopan in Recurrent C3 Glomerulopathy Following Kidney Transplant: A Report of Two Cases,” Kidney Medicine 6, no. 6 (2024): 100823. 38741947 10.1016/j.xkme.2024.100823 PMC11089394

